# Overview of rehabilitation interventions for ESPEN/EASO-defined sarcopenic obesity: a scoping review

**DOI:** 10.1007/s41999-025-01320-x

**Published:** 2025-10-16

**Authors:** Kosuke Suzuki, Masateru Hayashi, Yuichi Isaji, Hirotada Maeda, Katsuyoshi Tanaka, Takafumi Nasu, Koki Sasaki, Hayato Kunihara, Daisuke Sasaki, Wataru Okuyama, Yasuyuki Kurasawa, Takao Kaneko, Takashi Kitagawa

**Affiliations:** 1 Department of Rehabilitation, Yamagata Saisei Hospital, 79-1 Oki-Machi, Yamagata, 990-8545 Japan; 2 Department of Rehabilitation, Matsuoka Orthopedic Surgery and Internal Medicine Rehabilitation, 2-12-6 Higashikinpou, Gifu, 500-8167 Japan; 3 Department of Physical Therapy, School of Health Sciences, Bukkyo University, 7 Nishinokyo, Kyoto, 604-8418 Japan; 4 Department of Rehabilitation, Kanazawa Medical University Hospital, 1-1 Daigaku, Uchinada, 920-0293 Japan; 5 Department of Rehabilitation, Juko Osu Hospital, 2-17-5 Matubara, Nagoya, 460-0017 Japan; 6 Department of Rehabilitation, Hayashi Hospital, 1-3-5 Fuchu Echizen, Fukui, 915-8511 Japan; 7 Division of Rehabilitation, Musashino Emergency Hospital, 1-24-1 Kodaira, Tokyo, 187-0031 Japan; 8 Department of Rehabilitation, Iwami Medical Clinic, 7-1 Ekimae, Masuda, 698-0024 Japan; 9 Department of Rehabilitation, Tsukada Orthopedics, 3-9-20 Sakuramachi, Tsuchiura, 300-0037 Japan; 10 Department of Rehabilitation, Faculty of Health Sciences, Nagano University of Health and Medicine, 11-1 Imaihara, Nagano, 381-2227 Japan; 11 Department of Rehabilitation, Yamagata Prefectural Central Hospital, 1800 Aoyagi, Yamagata, 990-2292 Japan; 12 Department of Physical Therapy, School of Health Sciences, Shinshu University, 3-1-1 Asahi, Matsumoto, 390-8621 Japan

**Keywords:** Sarcopenic obesity, Rehabilitation, Scoping review, Exercise, Nutritional intervention

## Abstract

**Purpose:**

Sarcopenic obesity (SO) is a global concern characterized by the co-existence of sarcopenia and obesity. Although various interventions are recommended for SO, inconsistent definitions represent a barrier to clinical practice. This scoping review aimed to provide an overview of rehabilitation interventions for SO based on the definitions provided by the European Society for Clinical Nutrition and Metabolism and the European Association for the Study of Obesity.

**Methods:**

A systematic search was conducted across nine databases, with a final search conducted on February 27, 2024. The eligible studies included randomized controlled trials, prospective and retrospective cohort studies, and case–control studies without restrictions on language, country, sex, or publication date. The risk-of-bias assessment focused on muscle strength, physical function, and body composition. The risk of bias was assessed using the Risk of Bias 2 tool for randomized trials and the Newcastle–Ottawa scale for non-randomized studies.

**Results:**

Four randomized controlled trials involving 246 participants were included, all of which were conducted in communities. In addition, three studies comprised exclusively female participants, and all four studies employed resistance exercises. One study used a combination of resistance exercises and nutritional interventions. The overall risk of bias was high for all outcomes.

**Conclusion:**

The reported studies emphasized the limited number of studies covering diverse populations and settings. Further studies with improved methodological rigor are essential to identify effective rehabilitation strategies for SO.

**Registration:**

The study protocol was registered in the Open Science Framework (https://osf.io/wak9n/) on January 31, 2024.

**Supplementary Information:**

The online version contains supplementary material available at 10.1007/s41999-025-01320-x.

## Key summary points

**Aim:**

This scoping review aimed to identify studies on sarcopenic obesity (SO) that followed the European Society for Clinical Nutrition and Metabolism (ESPEN) and the European Association for the Study of Obesity (EASO) definition and to present an overview of rehabilitation interventions, including resistance exercise, aerobic exercise, and nutritional interventions for SO.

**Findings:**

Four randomized controlled trials met the inclusion criteria, all of which were community-based and utilized resistance exercise as an intervention. Although these studies suggested improvements in physical function, muscle strength, and body composition, none measured quality of life, and all were assessed as having a "high" risk of bias.

**Message:**

This scoping review revealed the limited number of studies, their high risk of bias, and a lack of diversity in participants and interventions in the studies on SO. Therefore, rigorous research, including quantitative systematic reviews and meta-analyses, is needed to establish effective rehabilitation strategies for SO based on the ESPEN/EASO consensus definition.

**Supplementary Information:**

The online version contains supplementary material available at 10.1007/s41999-025-01320-x.

## Introduction

Sarcopenic obesity (SO) is a global concern characterized by the co-existence of sarcopenia and obesity [1]. SO is associated with multiple adverse health effects, including significantly poorer mental health and quality of life (QOL) compared to those in healthy individuals [2], a higher risk of adverse musculoskeletal outcomes compared to that in either sarcopenia or obesity [3], and a significantly increased risk of all-cause mortality compared to that in individuals without SO [4, 5]. In addition, 11% of older adults are affected by SO [6], which is expected to increase due to age-related decreases in skeletal muscle mass [7] and increases in body fat [8]. These findings highlight the urgent need to address this concern.

The treatment strategies for SO vary; resistance exercises, aerobic exercises, and nutritional interventions have been recommended [9–11]. Exercise [12–14] and nutritional interventions [14, 15] have been shown to improve body composition and physical function and are considered effective interventions for SO. Various interventions have been recommended, but inconsistencies in the definition of SO have been a barrier to clinical practice.

The definition of SO remains unclear because of inconsistencies in existing definitions [9, 11]. Several systematic reviews on exercise interventions for SO exist; a notable limitation of these studies is the variability in definitions [13, 16–20]. In addition, the prevalence of SO varies widely depending on the diagnostic criteria used [6, 21, 22], indicating high heterogeneity. The definition of SO was established in 2022 by the European Society for Clinical Nutrition and Metabolism (ESPEN) and the European Association for the Study of Obesity (EASO) in a Consensus Statement on the definition and diagnostic criteria [1]. The ESPEN/EASO defines three levels of diagnostic procedures: screening, diagnosis, and staging. These steps are crucial for diagnosing medical conditions. The ESPEN/EASO definition recommends confirming the diagnosis through further testing after a positive screening result. The ESPEN/EASO definition is expected to be increasingly adopted in future studies because of its consistency. However, few intervention studies have been reported based on the current definition of SO as defined by ESPEN/EASO.

Owing to the recent definition by ESPEN/EASO and the limited number of studies, whether a meta-analysis can be performed is questionable. Therefore, a scoping review (ScR) should be conducted as a first step. A key advantage of the ScR is that mapping facilitates the identification of critical research gaps. This facilitates the formulation of recommendations to promote standardization across the entire domain and minimizes research waste [23]. This review aimed to identify studies on SO that followed the ESPEN/EASO definition and to present an overview of rehabilitation interventions, including the recommended resistance exercise, aerobic exercise, and nutritional interventions for SO.

## Methods

This study was conducted using the Preferred Reporting Items for Systematic Reviews and Meta-analyses extension for a scoping review (PRISMA-ScR) [24] and the Joanna Briggs Institute (JBI) methodology [25]. The study protocol was registered in the Open Science Framework (https://osf.io/wak9n/) on January 31, 2024. The requirement for ethical approval of the study was waived as this study was based on the data of previous studies. The participants, concept, and context framework were used to define the inclusion criteria.

### Participants

This study included individuals with SO as defined by the ESPEN/EASO. Individuals with comorbidities were also included because the ESPEN/EASO definition determines staging based on the occurrence of comorbidities. Age was not restricted. Patients with dynapenia and obesity due to presarcopenia were excluded. Moreover, studies in which all participants met the inclusion criteria were included, and studies in which only a subset of participants met the criteria were excluded.

### Concept

We included studies focused on rehabilitation combined with nutritional intervention or nutritional intervention alone. Interventions involving only medications were excluded.

This study measured several outcomes, including physical function (walking speed, Short Physical Performance Battery test, Timed Up and Go test, and single leg stance test), muscle strength (knee extension, grip strength, and sit-to-stand test), body composition (body fat mass, skeletal muscle mass, body mass index, body fat percentage, abdominal circumference, and weight), and QOL (Medical Outcomes Study Short Form 36 and EuroQol-5 Dimension Questionnaire).

The ESPEN/EASO definition [1] includes three steps: screening, diagnosis, and staging. It states that a diagnosis should be made after a positive screening result. Therefore, we included studies that fulfilled both screening and diagnostic criteria. If a limited number of such studies were applicable, we selected the studies that applied the diagnostic criteria. Ethnicity-specific criteria were adopted directly as presented in the original studies, based on information provided in their online supplementary materials [1].

### Context

No restrictions were set on the setting, sex, race, country, language, follow-up period, or publication date of the study.

### Type of sources

This study included randomized controlled trials (RCTs), crossover RCTs, cluster RCTs, quasi-RCTs, non-RCTs, prospective and retrospective cohort studies, and case–control studies. Case reports, case series, cross-sectional studies, systematic reviews, meta-analyses, narrative reviews, study protocols, and conference abstracts were excluded. Protocols and conference abstracts were included in the primary screening, and secondary screening was performed to identify published articles.

### Search strategy

The search strategy aimed to identify both published and unpublished studies. A systematic search was conducted using the following databases: MEDLINE via PubMed, Cochrane Central Register of Controlled Trials, Embase via ProQuest Dialog, Cumulative Index to Nursing and Allied Health Literature via EBSCOhost, Web of Science, Physiotherapy Evidence Database, and OpenGrey as a source of grey literature. In addition, we searched for ongoing trials in the following trial registers: the World Health Organization International Clinical Trials Registry Platform and ClinicalTrials.gov. The titles and abstracts of the relevant articles and the index terms used to describe them were used to create a search strategy for the nine databases. Previous studies [14, 20, 26, 27] were also consulted for reference (Online Resource 1). The last search date was February 27, 2024.

### Study selection and data extraction

All identified citations were collated and uploaded to Rayyan (Qatar Computing Research Institute, Ar Rayyan, Qatar), and duplicates were removed [28]. Following a pilot test, the titles and abstracts were screened by independent reviewers (HM, KT, YI, and TN) to assess the inclusion criteria for the review. Independent reviewers (TKa, KSa, MH, HK, and KSu) assessed the full text of the selected citations in detail based on the inclusion criteria. The reasons for the exclusion of the sources of evidence in the full text that did not meet the inclusion criteria were recorded. Any disagreements between the reviewers at each stage of the selection process were resolved through discussion or by an additional reviewer (TKi). The search results and study inclusion process are reported in a PRISMA 2020 flow diagram [29]. Independent reviewers (YI, MH, TN, and KSu) used Microsoft Excel (Microsoft Corporation, 2019, US) to extract data from the papers included in the ScR. The extracted data included author(s), year of publication, origin or country of origin, setting, study design, sample size, follow-up period, participant characteristics [age, sex, diagnostic criteria (sarcopenia and obesity), and presence of complications], exercise characteristics (type of intervention, frequency, intensity, number of sets, and duration), outcomes (indicators used, whether the outcome improved, and key findings), adherence (number of participants and dropout rate), and study limitations. For a multi-arm study, the data for the intervention groups were extracted separately. Any disagreements between the reviewers were resolved through discussion or by an additional reviewer (TKi). If appropriate, the authors of the papers were contacted to request missing or additional data, where required.

### Assessment of risk of bias

Independent reviewers (DS and WO) assessed the risk of bias. The Risk of Bias 2 tool [30] was used for RCTs and the Newcastle–Ottawa scale for non-RCTs [31]. Disagreements between the reviewers were discussed, and in case of a lack of consensus, a third reviewer (KSu) made the decision.

### Data analysis and presentation

Outcomes identified in the literature were analyzed according to four categories: physical function, muscle strength, body composition, and QOL.

## Results

### Study selection

**Fig. 1:**
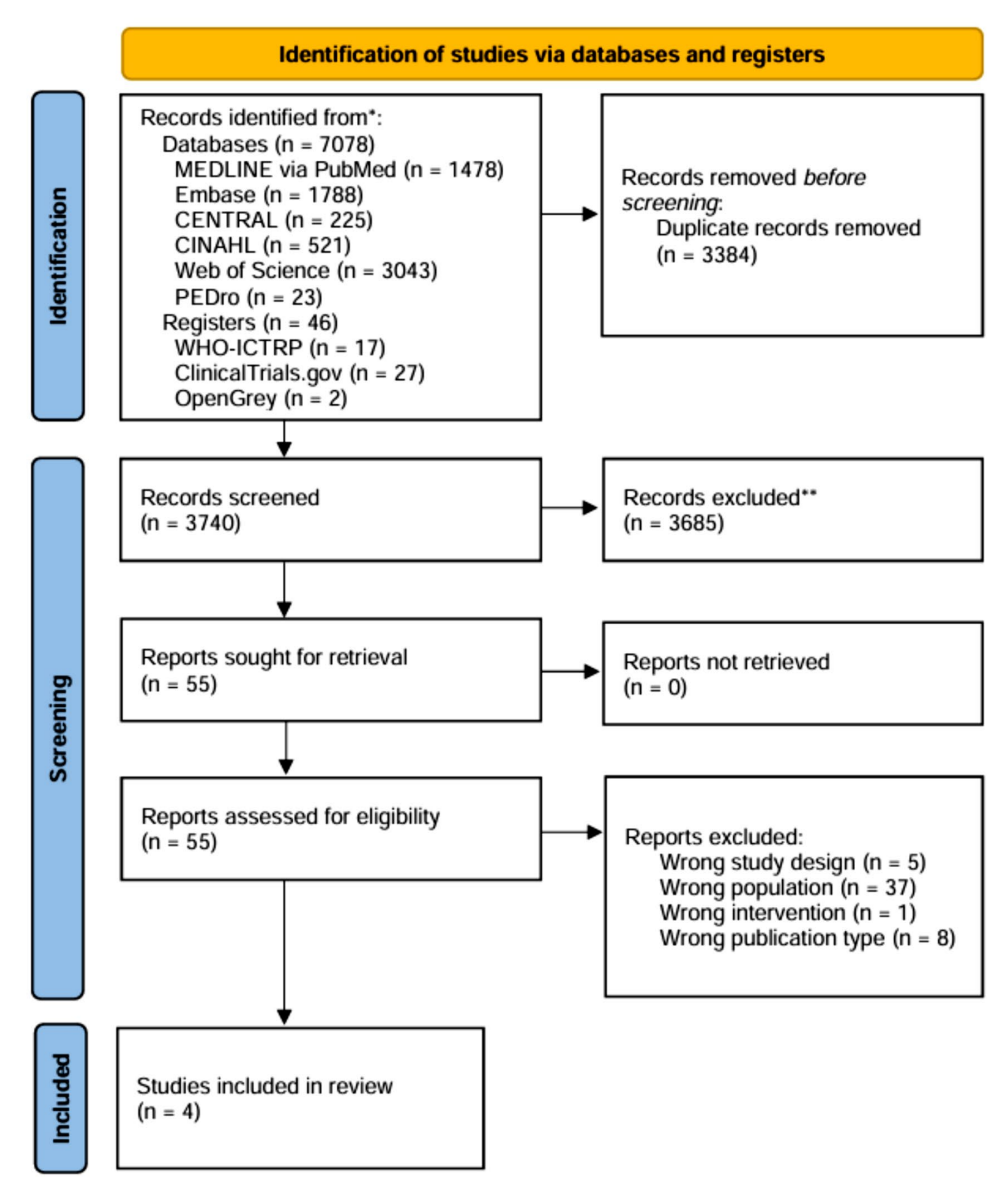
Preferred reporting items for systematic reviews and meta-analyses (PRISMA) flow diagram. *CENTRAL* Cochrane Central Register of Controlled Trials, *CINAHL* Cumulative Index to Nursing and Allied Health Literature, *PEDro* Physiotherapy Evidence Database, *WHO-ICTRP* the World Health Organization International Clinical Trials Registry Platform

The database and registry searches identified 7124 records. Subsequent de-duplication removed 3384 records. After title and abstract screening, 3685 records were excluded, and 55 reports were subjected to full-text screening. Ultimately, four studies met the inclusion criteria (Fig. 1), and 51 reports were excluded by full-text screening; the reasons for their exclusion are presented in Online Resource 2.

### Study characteristics

Table 1 summarizes the characteristics of the included studies. Four RCTs were included, and the intervention setting was the community in all four studies. One study met the screening and diagnostic criteria [32], and three met only the diagnostic criteria [10, 33, 34]. The sample sizes ranged from 17 to 139. These studies were conducted in the United States, Japan, Taiwan, and Iran.

**Table 1:** Characteristics of included studies

Author, year	Country	Setting	Study design	Sample size	Follow-up period	Age, mean (SD)	Female (%)
Balachandran et al. [32]	United States	Local community	RCT	17 (exercise: 8, control: 9)	15 weeks	IG: 71.6 (7.8) CG: 71 (8.2)	IG: 100 CG: 88.9
Kim et al. [10]	Japan	Urban community	RCT	139 (exercise + nutrition: 36, exercise: 35, nutrition: 34, control: 34)	12 weeks	Exercise + nutrition: 80.9 (4.2) Exercise: 81.4 (4.3) Nutrition: 81.2 (4.9) CG: 81.1 (5.1)	100
Lee et al. [33]	Taiwan	Local community	RCT	27 (exercise: 15, control: 12)	12, 36 weeks	IG: 70.1 (4.4) CG: 71.8 (5.2)	100
Banitalebi et al. [34]	Iran	The community	RCT	63 (exercise: 32, control: 31)	12 weeks	IG: 64.1 (3.8) CG: 64.1 (3.4)	100

*CG* control group, *IG* intervention group, *RCT* randomized controlled trial, *SD* standard deviation

Table 2 shows the diagnostic criteria used for sarcopenia and obesity and the presence of complications. Three studies employed the European Working Group on Sarcopenia in Older People criteria for sarcopenia [32–34], whereas one used the skeletal muscle index, grip strength, and walking speed [10].

**Table 2:** Diagnostic criteria and types of complications of sarcopenia and obesity

Author, year	Diagnostic criteria		Complications
	Sarcopenia	Obesity	
Balachandran et al. [32]	EWGSOP 2010	BMI ≥ 30 kg/m^2^	NR
Kim et al. [10]	SMI (DXA) < 5.67 kg/m^2^, grip strength < 17.0 kg, walking speed < 1.0 m/s	BF% ≥ 32% (DXA)	Hypertension, hyperlipidemia, diabetes, hip osteoarthritis, knee osteoarthritis
Lee et al. [33]	EWGSOP 2010	BF% > 35% (DXA)	NR
Banitalebi et al. [34]	EWGSOP2 2018	BF% > 32%, BMI > 30 kg/m^2^	Osteoporosis

*EWGSOP* European Working Group on Sarcopenia in Older People, *SMI* skeletal muscle index, *BMI* body mass index, *BF%* body fat percentage, *DXA* dual-energy X-ray absorptiometry, *NR* not reported

In terms of obesity criteria, two studies used body fat percentage [10, 34], one study used body mass index (BMI) [32], and one study used both BMI and body fat percentage [33]. Regarding complications, one study included osteoporosis [33], whereas the other included hypertension, hyperlipidemia, diabetes, hip osteoarthritis, and knee osteoarthritis [10]. Two studies did not report complications [32, 34].

Table 3 presents the details of the interventions. Two studies employed resistance training in the intervention groups [33, 34], while one employed fast circuit training [32]. The remaining study involved a combination of resistance training and aerobic training (exercise) in conjunction with amino acid and tea catechin supplementation (nutrition) as well as exercise-alone and nutrition-alone interventions [10]. The intervention was conducted for 12–15 weeks. The level of adherence was between 71 and 81% in the intervention group and between 81.3 and 85% in the control group [32–34]. One study reported combined data for these groups, resulting in an overall adherence rate of 98.6% [10]. The reported adverse events include fibromyalgia, shoulder pain, knee pain, and muscle soreness [32, 33].

**Table 3:** Intervention protocol details of the included studies

Author, year	Intervention	Frequency of intervention	Duration of intervention	Adherence	Adverse events
Balachandran et al. [32]	High-speed circuit training	2 days/1 week	15 weeks	IG: 81%, CG: 85%	IG: fibromyalgia (*n* = 1) CG: shoulder pain (*n* = 1)
Kim et al. [10]	Exercise + nutrition, exercise, nutrition	Exercise: 2 days/1 week Health education: once every 2 weeks	12 weeks	Total: 137/139 (98.6%)	None
Lee et al. [33]	Individual resistance exercises	3 days/1 week	12 weeks	100%	None
Banitalebi et al. [34]	Resistance training using elastic tubes or elastic bands	3 days/1 week	12 weeks	IG: 71.0%, CG: 81.3%	Shoulder pain Knee pain Muscle soreness

*IG* intervention group, *CG* control group

Table 4 presents the measured outcomes, key outcome findings, and limitations of the study. QOL was not measured in any of the four studies. In terms of limitations, three studies indicated that the sample size was insufficient [32–34] and that the participants were exclusively women [10, 33, 34].

**Table 4:** Outcomes and outcome key findings measured in the included studies and study limitations

Author, year	Outcome (physical function)	Outcome (muscle strength)	Outcome (body composition)	Outcome key findings	Limitations of the study
Balachandran et al. [32]	SPPB (modified)	Peak power (leg press and chest press) 1 RM (leg press and chest press) GS	BF%, LBM, SMI	The high-speed circuit-training intervention resulted in a notable enhancement in the modified SPPB score, with a considerable effect size. In contrast, the traditional strength/hypertrophy training group did not demonstrate a significant change	Small sample size, the use of study-specific cut-off scores for SPPB, and use of BIA to identify sarcopenia
Kim et al. [10]	Usual walking speed	GS, knee extension strength	BMI, muscle mass, body fat mass, BF%, SMI	The combination of exercise and nutrition was effective in improving body fat, blood components, and physical function in older women with sarcopenic obesity. However, no additive effects of the combination intervention could be confirmed	The effect of nutritional supplementation on placebo is unknown, the separate effects of aerobic and resistance exercise are unknown, confusion between the use of DXA and BIA, only women were included
Lee et al. [33]	FFR, SLS, TUG test	GS, TCR test	Total body fat, total BF%, ALM, LMI (whole-body skeletal muscle mass/height), SMI (ALM/height)	Significant improvements have been made in physical capacity parameters, namely FFR, GS, TUG-test scores, and TCR-test scores. The improvements in GS, TUG-test scores, and TCR-test scores were more significant in the intervention group than in the control group; however, the TUG-test scores were not significantly different	Small sample size, only women were included, and the effect of exercise on the BMD/T-score of the hip was not assessed
Banitalebi et al. [34]	TUG test, 6MWT, 10MWT	30-s chair stand test	Body mass, BF%, BMI, WHR, WC	In addition to potentially reducing fall risk and reversing sarcopenia, resistance exercise has been demonstrated to improve cardiometabolic function and reduce the risk of developing type-2 diabetes and cardiovascular disease	Only women were included, small sample size, short intervention period, lack of *V*O_2_max measurement, and the dropout rate

*SPPB* Short Physical Performance Battery test, *FFR* functional forward reach, *SLS* single-leg stance, *TUG test* Timed Up and Go test, *6MWT* 6-min walk test, *10MWT* 10-m walk test, *GS* grip strength, *TCR* test, timed chair rise test, *BF%* body fat percentage, *LBM* lean body mass, *SMI* skeletal muscle index, *ALM* appendicular lean mass, *LMI* lean muscle mass index, *WHR* waist-hip ratio, *WC* waist circumference, *DXA* dual-energy X-ray absorptiometry, *BIA* bioelectrical impedance analysis, *BMD/T-score* bone mineral density T-score, *BMI* body mass index, *RM* repetition maximum, *VO*_*2*_*max* maximum oxygen uptake

### Risk of bias in included studies

**Fig. 2:**
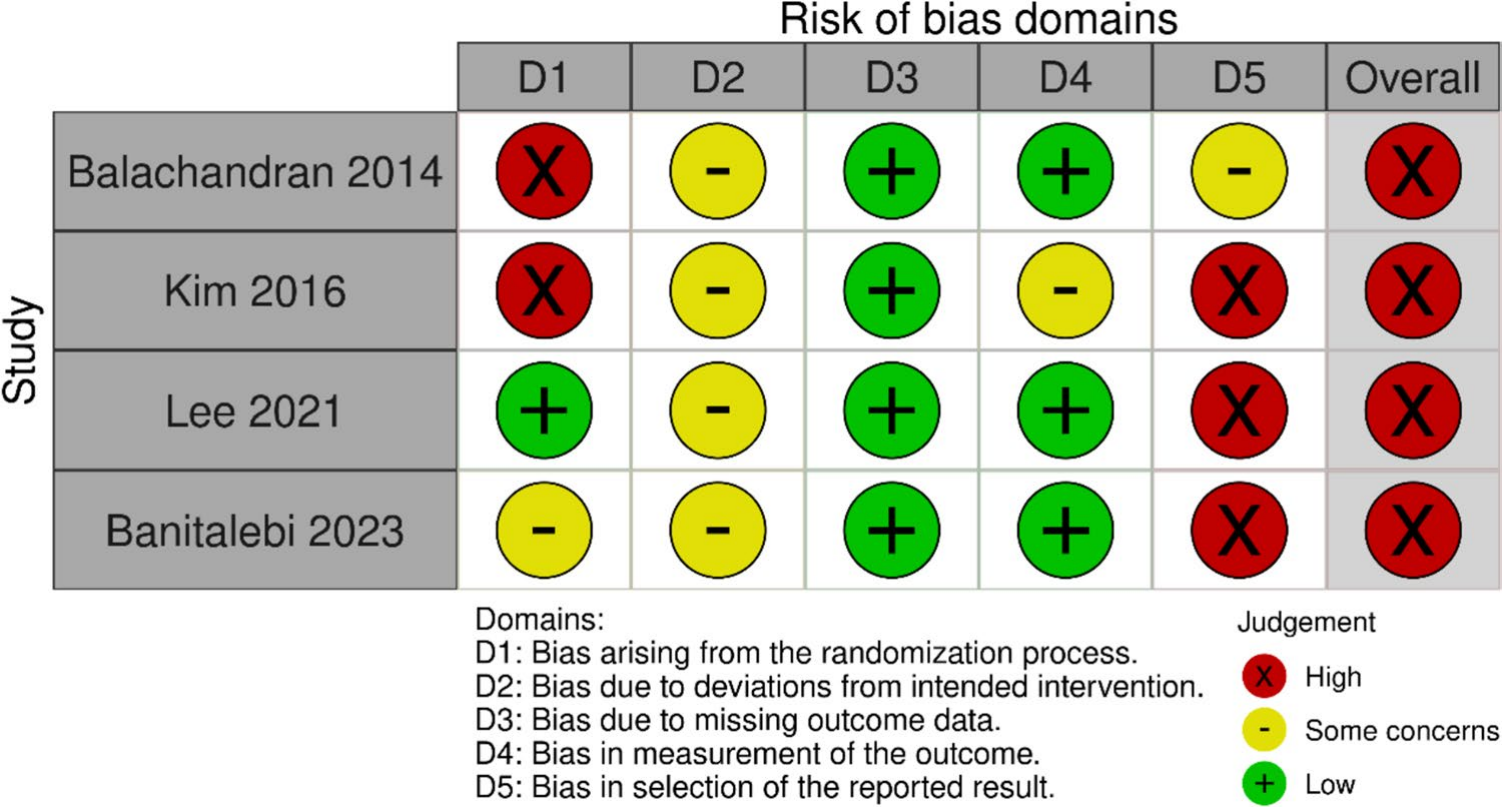
Risk of bias for physical function, muscle strength, and body composition

The results of the risk-of-bias assessment are shown in Fig. 2. As all four studies included in this review were RCTs, only the Risk of Bias 2 tool was used. The results of the risk-of-bias assessment for each outcome in the three categories of physical function, muscle strength, and body composition were consistent, with an overall risk-of-bias rating of “high” in all four studies.

## Discussion

This study is the first review to identify studies on SO according to the ESPEN/EASO definition and presents an overview of rehabilitation interventions for SO, including recommended resistance exercises, aerobic exercises, and nutritional interventions. The number of studies included in this review was limited to four, which may restrict the generalizability of the findings. Additionally, the settings, intervention contents, and target populations were limited. Furthermore, two studies did not describe complications [32, 34], and the effect of the intervention in cases with comorbidities was unknown. In a broad literature review on nutritional management and physical activity for SO [35], many studies targeted men. Based on the results of this study, a bias may exist in the participants in the reports on SO based on the ESPEN/EASO definition. The participants of the studies included in this review were almost all women, and whether the same results would be obtained in men is unclear. Sarcopenia has various causes, such as hospital-related, acute, and ectopic sarcopenias, and the etiopathogenesis differs [36]. Inflammation has also been shown to be associated with SO [11]. Thus, differences may exist in responsiveness to interventions for acute inflammation in hospital environments or among residents of care facilities. Therefore, the results were not the same for those reported in hospital environments and in residents of care facilities. Focusing on complications, an umbrella review of SO suggested that cognitive impairment, coronary artery disease, and dyslipidemia may be risk factors for SO [37]. Furthermore, a review of the prevalence of SO examined various diseases and suggested that the prevalence of SO might increase with comorbidities [38]. However, only two studies included patients with complications, and one of these studies included patients with dyslipidemia but did not include patients with cognitive impairment or coronary artery disease. This indicates that investigating the effects of rehabilitation interventions for a wide range of diseases that target SO, as defined by the ESPEN/EASO, is necessary.

Focusing on the intervention content, all four studies included in this review used resistance exercises. In addition, all four studies tended to improve the physical function outcomes. A bibliometric analysis suggested that resistance exercise or a combination of resistance exercise and nutritional intervention, mainly protein supplementation, may be promising [39]. This finding is consistent with the results of the present study. Furthermore, the ScR on the effectiveness of exercise interventions for SO broadly suggests that resistance exercise may be more effective than aerobic exercise or combined exercise therapy with resistance and aerobic exercises in improving muscle mass [40]. In this study, the efficacy of interventions such as aerobic and multi-component exercises remains to be elucidated through rigorous research. This is because aerobic and multi-component exercises have the potential to reduce not only fat but also muscle, in addition to weight loss [41, 42]. Consequently, these interventions are anticipated to be less frequently implemented compared with resistance exercise, particularly in complex conditions such as SO. All four studies involved resistance exercises; therefore, whether the same results would be obtained for SO, as defined by ESPEN/EASO, is unclear. Therefore, further studies that include men and cover a variety of diseases, settings, and interventions need to be conducted.

Considering the risk of bias, two studies were classified as high risk in the randomization process (domain 1), and three studies were classified as high risk in the reporting of outcomes (domain 5). During the randomization process, an absence of allocation concealment was identified. Furthermore, issues were identified in the outcome-reporting process, including the lack of protocol documentation and comparisons of outcomes that differed from those specified in the protocol. Considering these findings, future studies should address the current challenges by ensuring allocation concealment prior to intervention assignment and by preregistering study protocols and adhering to them throughout the trial.

The strength of this study is that it identified the content of rehabilitation interventions and clinical and methodological concerns related to SO as defined by the ESPEN/EASO. The interventions identified in this study can be used as a reference for similar target populations. In addition, the treatment effects in more target populations could be clarified by modifying the clinical and methodological issues identified in this study. In addition, we exhaustively searched six databases and three registries without any limitations regarding language, year of publication, or other factors. Furthermore, the search was conducted using a rigorous methodology based on PRISMA-ScR and JBI. In addition, we used a rigorous methodology with predeveloped protocols.

### Limitations of the review

This review has few limitations. First, the focus was on rehabilitation interventions; pharmacotherapy and bariatric surgery were excluded. Consequently, interventions for SO, as defined by ESPEN/EASO, are only partially covered. Second, this study was an ScR, and no quantitative analysis, such as a meta-analysis, was conducted, making it challenging to interpret the combined results of the four studies.

### Limitations of included studies

Regarding the intervention content, none of the studies included in the present analysis involved dietary interventions incorporating caloric restriction. As lifestyle interventions, including dietary weight loss, have also been suggested to be effective for SO [11], this approach warrants further investigation in future studies. With respect to outcomes, a limitation of the four included studies is the absence of QOL assessment. Previous studies have shown that individuals with SO have lower QOL [43]. Clarifying the impact of rehabilitation interventions on QOL in individuals with SO, as defined by ESPEN/EASO, is considered necessary from the perspective of treatment selection and healthcare policy. Although all four studies demonstrated an inclination towards improved outcomes, the results should be interpreted cautiously, considering the "high" risk of bias. Quantitative systematic reviews and meta-analyses are recommended considering these factors.

## Conclusion

This ScR aimed to provide an overview of rehabilitation interventions for SO as defined by ESPEN/EASO. The number of studies included in this review was limited to four, which limited the setting, intervention content, and target population. Furthermore, a notable limitation was the lack of QOL assessment in the included studies. Focusing on the intervention content, all four studies included in this review used resistance exercises. However, the risk of bias was assessed as “high” in all four studies; therefore, the results should be interpreted cautiously. Further studies are needed to investigate various diseases, settings, and interventions in male participants. In addition, quantitative systematic reviews and meta-analyses are warranted.

## Supplementary Information

Below is the link to the electronic supplementary material.

Supplementary file1 (DOCX 39 KB)

## Data Availability

The datasets generated and/or analyzed during the current study are available from the corresponding author on reasonable request.
