# Supplementary material for: Overview of rehabilitation interventions for ESPEN/EASO-defined sarcopenic obesity: a scoping review
Source: Eur Geriatr Med. 2025 Oct 16;17(2):685–95. doi: 10.1007/s41999-025-01320-x (PMC13109205; doi:10.1007/s41999-025-01320-x)
Supplement: Supplementary file 1 — Supplementary file1 (DOCX 39 KB) [file 41999_2025_1320_MOESM1_ESM.docx]

Table A.1. Preferred Reporting Items for Systematic reviews and Meta-Analyses extension for Scoping Reviews (PRISMA-ScR) Checklist

| **SECTION** | **ITEM** | **PRISMA-ScR CHECKLIST ITEM** | **REPORTED ON PAGE #** |
| --- | --- | --- | --- |
| **TITLE** | | | |
| Title | 1 | Identify the report as a scoping review. | Title |
| **ABSTRACT** | | | |
| Structured summary | 2 | Provide a structured summary that includes (as applicable): background, objectives, eligibility criteria, sources of evidence, charting methods, results, and conclusions that relate to the review questions and objectives. | Abstract |
| **INTRODUCTION** | | | |
| Rationale | 3 | Describe the rationale for the review in the context of what is already known. Explain why the review questions/objectives lend themselves to a scoping review approach. | 6-7 |
| Objectives | 4 | Provide an explicit statement of the questions and objectives being addressed with reference to their key elements (e.g., population or participants, concepts, and context) or other relevant key elements used to conceptualize the review questions and/or objectives. | 7 |
| **METHODS** | | | |
| Protocol and registration | 5 | Indicate whether a review protocol exists; state if and where it can be accessed (e.g., a Web address); and if available, provide registration information, including the registration number. | 8 |
| Eligibility criteria | 6 | Specify characteristics of the sources of evidence used as eligibility criteria (e.g., years considered, language, and publication status), and provide a rationale. | 8-10 |
| Information sources* | 7 | Describe all information sources in the search (e.g., databases with dates of coverage and contact with authors to identify additional sources), as well as the date the most recent search was executed. | 10 |
| Search | 8 | Present the full electronic search strategy for at least 1 database, including any limits used, such that it could be repeated. | Online Resource 1 |
| Selection of sources of evidence† | 9 | State the process for selecting sources of evidence (i.e., screening and eligibility) included in the scoping review. | 10-11 |
| Data charting process‡ | 10 | Describe the methods of charting data from the included sources of evidence (e.g., calibrated forms or forms that have been tested by the team before their use, and whether data charting was done independently or in duplicate) and any processes for obtaining and confirming data from investigators. | 10-11 |
| Data items | 11 | List and define all variables for which data were sought and any assumptions and simplifications made. | 11 |
| Critical appraisal of individual sources of evidence§ | 12 | If done, provide a rationale for conducting a critical appraisal of included sources of evidence; describe the methods used and how this information was used in any data synthesis (if appropriate). | 11-12 |
| Synthesis of results | 13 | Describe the methods of handling and summarizing the data that were charted. | 12 |
| **RESULTS** | | | |
| Selection of sources of evidence | 14 | Give numbers of sources of evidence screened, assessed for eligibility, and included in the review, with reasons for exclusions at each stage, ideally using a flow diagram. | Fig. 1 |
| Characteristics of sources of evidence | 15 | For each source of evidence, present characteristics for which data were charted and provide the citations. | 12-14 |
| Critical appraisal within sources of evidence | 16 | If done, present data on critical appraisal of included sources of evidence (see item 12). | 15, Fig. 2 |
| Results of individual sources of evidence | 17 | For each included source of evidence, present the relevant data that were charted that relate to the review questions and objectives. | 29-33 |
| Synthesis of results | 18 | Summarize and/or present the charting results as they relate to the review questions and objectives. | 29-33 |
| **DISCUSSION** | | | |
| Summary of evidence | 19 | Summarize the main results (including an overview of concepts, themes, and types of evidence available), link to the review questions and objectives, and consider the relevance to key groups. | 16-20 |
| Limitations | 20 | Discuss the limitations of the scoping review process. | 19-20 |
| Conclusions | 21 | Provide a general interpretation of the results with respect to the review questions and objectives, as well as potential implications and/or next steps. | 20 |
| **FUNDING** | | | |
| Funding | 22 | Describe sources of funding for the included sources of evidence, as well as sources of funding for the scoping review. Describe the role of the funders of the scoping review. | Funding |

JBI = Joanna Briggs Institute; PRISMA-ScR = Preferred Reporting Items for Systematic reviews and Meta-Analyses extension for Scoping Reviews.

* Where sources of evidence are compiled from, such as bibliographic databases, social media platforms, and Web sites.

† A more inclusive/heterogeneous term used to account for the different types of evidence or data sources (e.g., quantitative and/or qualitative research, expert opinion, and policy documents) that may be eligible in a scoping review as opposed to only studies. This is not to be confused with information sources (see first footnote).

‡ The frameworks by Arksey and O’Malley and Levac and colleagues and the JBI guidance refer to the process of data extraction in a scoping review as data charting.

§ The process of systematically examining research evidence to assess its validity, results, and relevance before using it to inform a decision. This term is used for items 12 and 19 instead of "risk of bias" (which is more applicable to systematic reviews of interventions) to include and acknowledge the various sources of evidence that may be used in a scoping review (e.g., quantitative and/or qualitative research, expert opinion, and policy document).

A. 1. Search strategy

**MEDLINE via PubMed search strategy**

Participant keywords: ((((Sarcopenia[MeSH Terms]) AND (Obesity[MeSH Terms])) OR ((((("sarcopenic obesity"[Title/Abstract]) OR (sarcobesity[Title/Abstract])) OR ("sarcopenic obese"[Title/Abstract])) OR ("obese sarcopenia"[Title/Abstract])) OR ((sarcopenia[Title/Abstract]) AND (obesity[Title/Abstract])))))

Intervention keywords: (((((((((((((((((((((Rehabilitation[MeSH Terms]) OR (Exercise[MeSH Terms])) OR (Physical Therapy Modalities[MeSH Terms])) OR (Physical Therapy Specialty[MeSH Terms])) OR (Diet[MeSH Terms])) OR (Nutrition Therapy[MeSH Terms])) OR (Electric Stimulation Therapy[MeSH Terms])) OR (train*[Title/Abstract])) OR (resistance*[Title/Abstract])) OR (exercis*[Title/Abstract])) OR (endurance[Title/Abstract])) OR (physical*[Title/Abstract])) OR ("electric stimulat*"[Title/Abstract])) OR ("whole-body electromyostimulat*"[Title/Abstract])) OR ("WB-EMS"[Title/Abstract])) OR (protein[Title/Abstract])) OR (supplement*[Title/Abstract])) OR ("amino acid"[Title/Abstract])) OR (rehabilitation[Title/Abstract])) OR (diet[Title/Abstract])) OR (nutrition therapy[Title/Abstract]))

**CENTRAL search strategy**

Participant keywords: (((([mh Sarcopenia]) AND ([mh Obesity])) OR ((((("sarcopenic obesity":ti,ab) OR (sarcobesity:ti,ab)) OR ("sarcopenic obese":ti,ab)) OR ("obese sarcopenia":ti,ab)) OR ((sarcopenia:ti,ab) AND (obesity:ti,ab)))))

Intervention keywords: ((((((((((((((((((((([mh Rehabilitation]) OR ([mh Exercise])) OR ([mh "Physical Therapy Modalities"])) OR ([mh "Physical Therapy Specialty"])) OR ([mh Diet])) OR ([mh "Nutrition Therapy"])) OR ([mh "Electric Stimulation Therapy"])) OR (train*:ti,ab)) OR (resistance*:ti,ab)) OR (exercis*:ti,ab)) OR (endurance:ti,ab)) OR (physical*:ti,ab)) OR (("electric" NEXT stimulat*):ti,ab)) OR (("whole-body" NEXT electromyostimulat*):ti,ab)) OR (WB-EMS:ti,ab)) OR (protein:ti,ab)) OR (supplement*:ti,ab)) OR ("amino acid":ti,ab)) OR (rehabilitation:ti,ab)) OR (diet:ti,ab)) OR ("nutrition therapy":ti,ab))

**Embase via ProQuest Dialog search strategy**

Participant keywords: (((MESH.EXACT.EXPLODE(sarcopenia) AND MESH.EXACT.EXPLODE(obesity)) OR (TI,AB(“sarcopenic obesity”) OR TI,AB(sarcobesity) OR TI,AB(“sarcopenic obese”) OR TI,AB(“obese sarcopenia”) OR (TI,AB(sarcopenia) AND TI,AB(obesity))))

Intervention keywords: (MESH.EXACT.EXPLODE(rehabilitation) OR MESH.EXACT.EXPLODE(exercise) OR MESH.EXACT.EXPLODE(“physical therapy modalities”) OR MESH.EXACT.EXPLODE(“physical therapy specialty”) OR MESH.EXACT.EXPLODE(diet) OR (MESH.EXACT.EXPLODE(“nutritional support”) OR MESH.EXACT.EXPLODE(“nutrition therapy”)) OR MESH.EXACT.EXPLODE(“electric stimulation therapy”) OR TI,AB(train*) OR TI,AB(resistance*) OR TI,AB(exercis*) OR TI,AB(endurance) OR TI,AB(physical*) OR TI,AB(“electric stimulat*“) OR TI,AB(“whole body electromyostimulat*“) OR TI,AB(WB-EMS) OR TI,AB(protein) OR TI,AB(supplement*) OR TI,AB(“amino acid”) OR TI,AB(rehabilitation) OR TI,AB(diet) OR TI,AB(“nutrition therapy”)))

**CINAHL via EBSCOhost search strategy**

Participant keywords: (((((MH Sarcopenia+)) AND ((MH Obesity+))) OR ((((((TI "sarcopenic obesity" OR AB "sarcopenic obesity")) OR ((TI sarcobesity OR AB sarcobesity))) OR ((TI "sarcopenic obese" OR AB "sarcopenic obese"))) OR ((TI "obese sarcopenia" OR AB "obese sarcopenia"))) OR (((TI sarcopenia OR AB sarcopenia)) AND ((TI obesity OR AB obesity))))))

Intervention keywords: ((((((((((((((((((((((MH Rehabilitation+)) OR ((MH Exercise+))) OR ((MH "Physical Therapy Modalities+"))) OR ((MH "Physical Therapy Specialty+"))) OR ((MH Diet+))) OR ((MH "Nutrition Therapy+"))) OR ((MH "Electric Stimulation Therapy+"))) OR ((TI train* OR AB train*))) OR ((TI resistance* OR AB resistance*))) OR ((TI exercis* OR AB exercis*))) OR ((TI endurance OR AB endurance))) OR ((TI physical* OR AB physical*))) OR ((TI "electric stimulat*" OR AB "electric stimulat*"))) OR ((TI "whole-body electromyostimulat*" OR AB "whole-body electromyostimulat*"))) OR ((TI WB-EMS OR AB WB-EMS))) OR ((TI protein OR AB protein))) OR ((TI supplement* OR AB supplement*))) OR ((TI "amino acid" OR AB "amino acid"))) OR ((TI rehabilitation OR AB rehabilitation))) OR ((TI diet OR AB diet))) OR ((TI "nutrition therapy" OR AB "nutrition therapy")))

**Web of Science search strategy**

Participant keywords: ((((Sarcopenia) AND (Obesity)) OR ((((("sarcopenic obesity") OR (sarcobesity)) OR ("sarcopenic obese")) OR ("obese sarcopenia")) OR ((sarcopenia) AND (obesity)))))

Intervention keywords: (((((((((((((((((((((Rehabilitation) OR (Exercise)) OR ("Physical Therapy Modalities")) OR ("Physical Therapy Specialty")) OR (Diet)) OR ("Nutrition Therapy")) OR ("Electric Stimulation Therapy")) OR (train*)) OR (resistance*)) OR (exercis*)) OR (endurance)) OR (physical*)) OR ("electric stimulat*")) OR ("whole-body electromyostimulat*")) OR (WB-EMS)) OR (protein)) OR (supplement*)) OR ("amino acid")) OR (rehabilitation)) OR (diet)) OR ("nutrition therapy"))

**PEDro search strategy**

Abstract & Title: ”sarcopenic obesity”

Method: clinical trial

**OpenGrey search strategy**

sarcopenic obesity

**WHO-ICTRP search strategy**

Condition: “sarcopenic obesity” OR sarcobesity

Intervention: rehabilitation OR exercise

Recruitment status: ALL

**ClinicalTraials.gov search strategy**

Condition/disease: “sarcopenic obesity” OR sarcobesity

Intervention/treatment: rehabilitation OR exercise OR physiotherapy

Table A.2. Reason for exclusion

| Author and year | Reason for　exclusion | Title |
| --- | --- | --- |
| Schoufour (2023) | wrong study design | Prevention Of Obesity, Sarcopenia And Sarcopenic Obesity In Retirement: Development Of Persuasive Technology To Stimulate Healthy Lifestyle Behaviour EResults Of The SO-NUTS Study |
| Teixeira (2023) | wrong study design | Inflammatory Biomarkers in Older Women with Obesity, Sarcopenia, and Sarcopenic Obesity |
| Vasileva (2021) | wrong study design | Rehabilitation with kinesiotherapy in muscle function and weight loss improving in obesity patients |
| Vasileva (2022) | wrong study design | INFLUENCE OF COMPLEX KINESIOTHERAPY ON METABOLISM IN CASES OF SARCOPENIC OBESITY |
| Vikram (2022) | wrong study design | Randomized controlled trial to compare the impact of resistance band exercises on change in skeletal muscle mass and function in individuals with sarcopenic obesity |
| Liu (2023) | wrong publication type | Effects of an Individualized Dietary Behavioural Change (IDBC) Programme and Exercise Training in Combination or Separately on Managing Sarcopenic Obesity in Community-dwelling Older Adults: A Randomized Controlled Trial |
| Camajani (2021) | wrong publication type | L-Leucine Supplementation for Preserving Lean Mass During Low Calorie Diet in Sarcopenic Obese Women: A Pilot Study |
| Gando (2017) | wrong publication type | Carotid Artery Parameters After Combined Exercise Training in Women with Sarcopenic Obesity |
| Hsu (2023) | wrong publication type | Hemodynamic response to different contractile speeds of resistance exercise in adults with sarcopenic obesity |
| Stoever (2015) | wrong publication type | Changes of Body Composition, Muscular Strength and Physical Performance Due to Resistance Training in Older Persons with Sarcopenic Obesity |
| Sweeney (2020) | wrong publication type | Ethnocentric differences in sarcopenicobesity and body composition in response to an aerobic and resistance exercise intervention for breast cancersurvivors |
| Ward (2011) | wrong publication type | Sarcopenia and sarcopenic obesity: Is it time the health system accepted fitness of older people as a health responsibility? |
| Wood (2023) | wrong publication type | Impact of EASO/ESPEN-defined Sarcopenic Obesity on Outcomes of Telehelath Weight Loss Program |
| Gadelha (2016) | wrong population | Effects of resistance training on sarcopenic obesity index in older women: A randomized controlled trial |
| Muscariello (2016) | wrong population | Dietary protein intake in sarcopenic obese older women |
| Vasconcelos (2016) | wrong population | Effects of a progressive resistance exercise program with high-speed component on the physical function of older women with sarcopenic obesity: a randomized controlled trial |
| Wittmann (2016) | wrong population | Impact of whole body electromyostimulation on cardiometabolic risk factors in older women with sarcopenic obesity: the randomized controlled FORMOsA-sarcopenic obesity study |
| Banitalebi (2020) | wrong population | Osteosarcopenic obesity markers following elastic band resistance training: A randomized controlled trial |
| Banitalebi (2021) | wrong population | Effect of 12-weeks elastic band resistance training on MyomiRs and osteoporosis markers in elderly women with Osteosarcopenic obesity: a randomized controlled trial |
| Camajani (2022) | wrong population | Very low calorie ketogenic diet combined with physical interval training for preserving muscle mass during weight loss in sarcopenic obesity: A pilot study |
| Chang (2020) | wrong population | Effect of resistance training on quality of life in older people with sarcopenic obesity living in long-term care institutions: A quasi-experimental study |
| Chen (2017) | wrong population | Effects of Different Types of Exercise on Body Composition, Muscle Strength, and IGF-1 in the Elderly with Sarcopenic Obesity |
| Chiu (2018) | wrong population | Effects of resistance training on body composition and functional capacity among sarcopenic obese residents in long-term care facilities: a preliminary study |
| Cunha (2018) | wrong population | The effects of resistance training volume on osteosarcopenic obesity in older women |
| El-Hak (2021) | wrong population | Efficacy of aerobic and core exercise training on improving muscle mass and physical performance in postmenopausal women with sarcopenic obesity |
| Ferhi (2023) | wrong population | Effects of Physical Activity Program on Body Composition, Physical Performance, and Neuromuscular Strategies during Walking in Older Adults with Sarcopenic Obesity: Randomized Controlled Trial |
| Gregori (2023) | wrong population | Indices of sarcopenic obesity are important predictors of finite element analysis-derived bone strength in older adults with obesity |
| Huang (2017) | wrong population | Body composition influenced by progressive elastic band resistance exercise of sarcopenic obesity elderly women: a pilot randomized controlled trial |
| Kemmler (2018) | wrong population | Effects of Combined Whole-Body Electromyostimulation and Protein Supplementation on Local and Overall Muscle/Fat Distribution in Older Men with Sarcopenic Obesity: The Randomized Controlled Franconia Sarcopenic Obesity (FranSO) Study |
| Kemmler (2018) | wrong population | Effect of whole-body electromyostimulation and / or protein supplementation on obesity and cardiometabolic risk in older men with sarcopenic obesity: the randomized controlled FranSO trial |
| Kemmler (2016) | wrong population | Whole-body electromyostimulation to fight sarcopenic obesity in community-dwelling older women at risk. Resultsof the randomized controlled FORMOsA-sarcopenic obesity study |
| Kemmler (2020) | wrong population | Safety of a Combined WB-EMS and High-Protein Diet Intervention in Sarcopenic Obese Elderly Men |
| Kemmler (2017) | wrong population | Whole-body electromyostimulation and protein supplementation favorably affect sarcopenic obesity in community-dwelling older men at risk: the randomized controlled FranSO study |
| Lee (2022) | wrong population | The protective role of dairy protein on sarcopenic obesity in middle-aged and older women: a community-based, 12-year, prospective cohort study |
| Liao (2018) | wrong population | Effects of elastic band exercise on lean mass and physical capacity in older women with sarcopenic obesity: A randomized controlled trial |
| Liao (2017) | wrong population | Effects of elastic resistance exercise on body composition and physical capacity in older women with sarcopenic obesity: A CONSORT-compliant prospective randomized controlled trial |
| Magtouf (2023) | wrong population | Influence of Concurrent Exercise Training on Ankle Muscle Activation during Static and Proactive Postural Control on Older Adults with Sarcopenic Obesity: A Multicenter, Randomized, and Controlled Trial |
| Maltais (2016) | wrong population | Effect of Resistance Training and Various Sources of Protein Supplementation on Body Fat Mass and Metabolic Profile in Sarcopenic Overweight Older Adult Men: A Pilot Study |
| Nabuco (2019) | wrong population | Effect of whey protein supplementation combined with resistance training on body composition, muscular strength, functional capacity, and plasma-metabolism biomarkers in older women with sarcopenic obesity: A randomized, double-blind, placebo-controlled trial |
| Ricardo (2016) | wrong population | Sarcopenia, Obesity, and Resistance Training |
| Jung (2022) | wrong population | Effects of Circuit Training Program on Cardiovascular Risk Factors, Vascular Inflammatory Markers, and Insulin-like Growth Factor-1 in Elderly Obese Women with Sarcopenia |
| Park (2017) | wrong population | Effects of 24-Week Aerobic and Resistance Training on Carotid Artery Intima-Media Thickness and Flow Velocity in Elderly Women with Sarcopenic Obesity |
| Qi (2023) | wrong population | Effects of water exercise on body composition and components of metabolic syndrome in older females with sarcopenic obesity |
| Sammarco (2017) | wrong population | Evaluation of Hypocaloric Diet With Protein Supplementation in Middle-Aged Sarcopenic Obese Women: A Pilot Study |
| Santos (2023) | wrong population | A dataset on skeletal muscle mass index, body composition and strength to determinate sarcopenia in bariatric patients |
| Stoever (2018) | wrong population | Influences of Resistance Training on Physical Function in Older, Obese Men and Women With Sarcopenia |
| Tannir (2022) | wrong population | Can Intentional Weight Loss Ameliorate Sarcopenia in Individuals with Obesity? A Longitudinal Interventional Study |
| Wood (2023) | wrong population | Impact of EASO/ESPEN-Defined Sarcopenic Obesity Following a Technology-Based Weight Loss Intervention |
| Yin (2023) | wrong population | Dietary behaviour change intervention for managing sarcopenic obesity among community-dwelling older people: a pilot randomised controlled trial |
| Zhou (2018) | wrong population | The Effects of Electrical Acupuncture and Essential Amino Acid Supplementation on Sarcopenic Obesity in Male Older Adults: a Randomized Control Study |
| Camajani (2022) | wrong intervention | Whey Protein, L-Leucine and Vitamin D Supplementation for Preserving Lean Mass during a Low-Calorie Diet in Sarcopenic Obese Women |
